# Equine Amplification and Virulence of Subtype IE Venezuelan Equine Encephalitis Viruses Isolated during the 1993 and 1996 Mexican Epizootics

**DOI:** 10.3201/eid0902.020124

**Published:** 2003-02

**Authors:** Dante Gonzalez, José G. Estrada-Franco, Anne-Sophie Carrara, Judith F. Aronson, Scott C. Weaver

**Affiliations:** * Instituto Nacional de Investigaciones Forestales, Agricolas y Pecuarias, Mexico City, Mexico; † University of Texas Medical Branch, Galveston, Texas, USA

**Keywords:** encephalitis virus, Venezuelan equine, encephalitis, horses, arboviruses, alphavirus, research

## Abstract

To assess the role of horses as amplification hosts during the 1993 and 1996 Mexican Venezuelan equine encephalitis (VEE) epizootics, we subcutaneously infected 10 horses by using four different equine isolates. Most horses showed little or no disease and low or nonexistent viremia. Neurologic disease developed in only 1 horse, and brain histopathologic examination showed meningeal lymphocytic infiltration, perivascular cuffing, and focalencephalitis. Three animals showed mild meningoencephalitis without clinical disease. Viral RNA was detected in the brain of several animals 12-14 days after infection. These data suggest that the duration and scope of the recent Mexican epizootics were limited by lack of equine amplification characteristic of previous, more extensive VEE outbreaks. The Mexican epizootics may have resulted from the circulation of a more equine-neurotropic, subtype IE virus strain or from increased transmission to horses due to amplification by other vertebrate hosts or transmission by more competent mosquito vectors.

*Venezuelan equine encephalitis virus* (VEEV; *Togaviridae*: *Alphavirus*) is an emerging pathogen of humans and equines in many parts of the New World (*1*–*3*). Sporadic outbreaks date to the 1930s or earlier in South America and have affected hundreds of thousands of people, horses, donkeys, and mules, causing high mortality rates in equines and severe illness in humans. Most outbreaks have been confined to northern South America, but one that began in Guatemala and El Salvador in 1969 spread northward through Mexico and reached Texas in 1971 (*2*,*4*,*5*). The etiologic agents during all of the major VEE outbreaks were subtype IAB or IC VEEV. Other subtypes of VEEV, including subtype IE strains that circulate in sylvatic and swamp habitats of Central America and Mexico, are traditionally considered enzootic, equine-avirulent, and incapable of exploiting horses as amplification hosts to cause widespread disease (*6*–*9*).

Before 1993, the only VEE outbreak confirmed by virus isolation in Mexico was the 1969–1971 epizootic/epidemic (*5*). The etiologic agent was a subtype IAB VEEV strain that likely originated from an inadequately inactivated equine vaccine preparation (*10*,*11*). Another VEE outbreak in Tamaulipas, Mexico, was detected serologically in 1965–1966, but no virus strains were isolated (*12*,*13*).

During 1993 and 1996, small outbreaks of equine encephalitis occurred near the Pacific Coast in the Mexican States of Chiapas and Oaxaca, respectively. These outbreaks involved 125 and 32 equine cases, respectively, with case-fatality rates of 50% and 38%; human VEE was not confirmed, although human seroprevalence in the region is high (J.G.E.-F., S.C.W., unpub. data). Antigenic, sequencing and phylogenetic studies indicated that the VEEV strains isolated from horses belong to subtype IE and are closely related to sylvatic, enzootic strains isolated nearby in sylvatic habitats on the Pacific Coast of Guatemala from 1968–1980 (*14*–*16*). These Mexican outbreaks represented the first confirmed equine cases attributed to VEEV subtype IE infection. Although the 1966 Tamaulipas epizootic may have been caused by subtype IE viruses that circulate nearby on the Gulf Coast of Mexico (*12*), the IE subtype was not known to be equine virulent (*8*) and had never been shown to be capable of equine amplification or of producing equine epizootics. The 1993 and 1996 Mexican epizootics placed these assumptions into doubt.

Partial sequence analysis of the PE2 envelope glycoprotein precursor gene of four isolates made during the 1993 and 1996 Mexican outbreaks showed that they are strongly linked to enzootic subtype IE VEEV isolates made in 1968 and 1980 along the Guatemalan Pacific Coast (*14*,*15*). Complete structural gene nucleotide sequence comparison of a Guatemalan 1968 isolate with a 1993 Mexican isolate showed that they differ by only 3% at the nucleotide level (*14*), and more recent genomic sequencing of four strains has shown an overall nucleotide sequence divergence of <2%. Only eight amino acid changes are predicted to have accompanied the emergence of the epizootic strains (*16*). This genetic similarity suggests that small numbers of mutations in enzootic progenitors may have resulted in acquisition of the equine-virulent phenotype, as indicated by previous studies of subtype IC VEEV emergence in Venezuela (*17*).

To determine the virulence and viremia characteristics of VEEV strains isolated during these Mexican outbreaks, and to assess retrospectively the role of equines as amplification hosts during epizootic transmission, we conducted experimental infections of horses with four different Mexican strains isolated from horses in 1993 and 1996. Although severe clinical neurologic disease occurred in one animal, low levels of viremia or none was detected after subcutaneous inoculation of any of the horses, which suggests that equines were not the principal amplification hosts in Mexico during the 1993 and 1996 epizootics.

## Methods

### Virus Preparation

The VEEV strains used for experimental infections are described in Table 1. These viruses (without further passage) were diluted in Eagle minimal essential medium (MEM) containing antibiotics and 10% antibody-negative normal horse serum before being used.

**Table 1:** Mexican equine isolates of *Venezuelan equine encephalitis virus* used for experimental infections

Strain	Date of isolation	Place of isolation	Tissue	Passage history^a^
OAX131	25 June 1996	Chahuites, Oaxaca State	Cerebrum	sm1, RK1
CPA201	29 June 1993	Rancho El Recuerdo, Mapastepec, Chiapas State; 15°, 25′ N 93° 01′ W	Brain	sm1, RK1
OAX142	5 July 1996	Tapanatepec, Oaxaca State	Cerebrum	sm1, RK1
I-290-93	12 July 1993	Rancho La Guadalupe, Mapastepec, Chiapas State 15°, 25′ N 93° 00′ W	Serum	sm1, RK1

^a^RK, rabbit kidney, sm, suckling mouse.

### Horse Infections

Ten antibody-negative horses were identified, ranging in estimated age from 24 to 36 months. Only horses with no evidence of preexisting alphavirus immunity were used in this study of experimental infections. One week before the VEEV inoculations, all horses were treated with insecticide to eliminate external parasites, and their temperature and blood counts were recorded 3 days before the viral inoculations. The horses were selected at random for inclusion in two experimental groups of six and four animals, respectively.

Horses were inoculated with four different Mexican subtype IE VEE epizootic isolates (Table 1). Two strains were obtained from the 1993 Chiapas outbreak and two others from the 1996 Oaxaca outbreak. The first set of six horses was inoculated (three each) with strain CPA201, isolated from the brain of an encephalitic horse during the 1993 Chiapas outbreak, and strain OAX 131, isolated from the brain of a diseased horse from the 1996 Oaxaca outbreak. The last group of four horses (two each) was inoculated with OAX 142, isolated from the brain of a moribund horse from Oaxaca in 1996, and the I-290-93 strain, from the serum of a diseased horse from the 1993 Chiapas outbreak.

All horses were infected by subcutaneous inoculation in the shoulder region of 0.6–1.0 mL of MEM containing 10% antibody-negative normal horse serum and 2,000 Vero cell PFU of VEEV, a dose comparable to that inoculated by alphavirus-infected mosquitoes (*18*). Although infection by the bite of an infected mosquito may potentiate viremia levels generated by some bunyaviruses (*19*,*20*), we used needle inoculation for two reasons: 1) almost all past experimental equine infections with other epizootic VEEV have been conducted with needle infections, and we wanted to generate data that could be compared directly to the literature; and 2) lack of a high containment insectary near the large animal biocontainment facility precluded the use of infected mosquitoes.

Each pair or trio of horses inoculated with a given virus strain was housed together in an isolation room within a large animal biocontainment building. Each horse was placed in a single stall and fed daily with fresh hay and water, and the horse’s clinical signs were monitored at least twice daily. Rectal temperature was recorded twice daily until day 14 postinoculation, beginning on the day before inoculation. Blood was collected twice daily by venipuncture until day 9 and serum samples were stored at -70°C for virus titration. Additional blood was also collected into EDTA-containing tubes every morning for up to 9 days, beginning the day before inoculation, and analyzed for hematocrit, hemoglobin determination, and platelet and leukocyte counts (also counts of basophils, eosinophils, and monocytes).

The maintenance and care of animals complied with the guidelines of the Centro Nacional de Investigaciones en Microbiologia, Instituto Nacional de Investigaciones Forestales, Agricolas y Pecuarias.

### Histopathologic Studies

All of the horses, except one (no. 4) that died on day 12, were killed 15 days after inoculation. Necropsies were carried out to extract tissues of interest. For histopathologic studies, samples were taken of white and gray matter from the anterior, median, and posterior brain. The medula oblongata and a portion of the spinal chord were taken at random. Tissue samples <5 mm in thickness were fixed in 10% neutral buffered formalin and processed by routine methods for paraffin embedding. Sections were stained with hematoxylin and eosin.

### Virus and Viral RNA Assays

Serum viremia levels were assayed by the inoculation of serial dilutions onto monolayers of Vero cells to assess plaque formation. Serial dilutions were also injected intracerebrally into 1- to 2-day-old mice, and titers were calculated as 50% lethal dose (LD_50_) values. Attempts to isolate VEEV from brain samples included the inoculation of Vero cell monolayers and inoculation of 1- to 2-day-old mice.

Reverse transcription–polymerase chain reactions (RT-PCR) were also carried out on RNA extracted from paraffin-embedded brain tissue from horses 1, 2, 3, 4, and 10 (insufficient brain tissue was left after histologic analyses on the remaining horses) to detect VEEV RNA. RNA was extracted from tissues by using the Qiagen (Valencia, CA) RNeasy kit according to the manufacturer’s protocol. Approximately 20 mg of each sample was placed in a microfuge tube with 1,200 μL of 100% xylene and vortexed to dissolve the paraffin. The tissue was washed twice with an equal volume of 100% ethanol, then dried and resuspended in 350 μL of the Qiagen RTL buffer before being ground with a pestle and 0.5 mm zirconia/silica beads (BioSpec Products, Bartlesville, OK). The RNA was eluted in 30 μL of Rnase-free water and 3 μL of RNasin (Promega, Madison, WI) and used for RT-PCR.

RT-PCR was performed with the OneStep RT-PCR kit (Qiagen). The primers were designed to amplify genome positions 8505–8882, yielding an expected 377-bp product: sense primer: 5′-CATAGACAATCCTGGTTACGACGAG-3′, reverse primer: 5′-CACCTGGCAAGCAGAAAGTATCC-3′. The 50-μL reaction mix was comprised of Qiagen OneStep RT-PCR buffer, 400 μM of each dNTP, 600 nM of each primer, and 10 μL of RNA sample. The samples, along with negative control (water alone) and positive control (viral RNA) samples, were placed in a thermocycler for: 30 min at 50°C, 15 min at 95°C, followed by 40 cycles at 94°C for 1 min, 56°C for 1 min, 72°C for 1 min, and a final extension of 10 min at 72°C. A seminested reaction followed with the same sense primer and a nested reverse primer 5′-GCACACCTGATGCACCTG-3′ to amplify genome positions 8505–8642, for an expected 137-bp product. For the seminested PCR, all initial PCR samples, including the controls, were diluted 1/50. The seminested PCR was performed by using *Taq* polymerase (Promega) for 30 cycles at 95°C for 30 sec, 56°C for 30 sec, 72°C for 2 min, and a final 72°C extension for 10 min.

PCR samples were analyzed on a 1% agarose gel, and visible DNA products were purified by using a QIAquick PCR purification kit (Qiagen) and sequenced by using the sense primer and the ABI PRISM Big Dye Terminator v3.0 kit (Applied Biosystems, Foster City, CA). Results were compared to the genomic viral sequences for the Mexican viruses used (GenBank Accession nos. AF448536, AF448537, and AF448538).

### Serologic Tests

Before the virus inoculation, serum samples from all horses were tested for preexisting antibodies to VEE, eastern (EEE), and western equine encephalitis (WEE) viruses by using an enzyme-linked immunosorbent assay (ELISA) with cell lysates prepared from BHK-21 cells infected with VEE virus strain Trinidad donkey (*21*,*22*), EEE virus strain 82V2137 (*23*), and WEE virus strain Fleming (*24*). After the horses were injected, seroconversion was detected by using Vero cell plaque reduction (>80%) neutralization tests (PRNT) with the CPA201 strain.

## Results

### Clinical Signs and Symptoms

**Figure 1:**
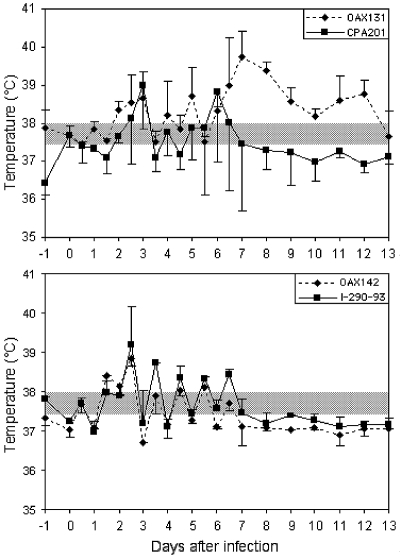
Mean rectal temperatures of equines infected with four different Mexican strains of subtype IE *Venezuelan equine encephalitis virus*. Bars indicate standard deviations; shaded box indicates approximate normal values.

All animals became infected by VEEV as indicated by seroconversion detected by PRNT (titers >1:40) on day 12 or 15 after inoculation. Symptoms of VEEV infection were absent in the infected horses, and only clinical signs were observed. In 9 of the 10 horses, the only clinical sign consistent with VEE was intermittent fever. Horses nos.1–5 and 10 showed a moderate temperature elevation of approximately 1°–2°C, whereas horses 6–8 and 9 showed only a small elevation of approximately 0.5°–1.0°C. Horse 6 did not display any febrile response, only normal variation in its temperature (Table 2). All animals had normal temperatures when they were killed on day 12 or 15. When it occurred, fever peaked 2–7 days after inoculation (Figure 1). Most horses displayed a slight anorexia, but none completely ceased eating during the study, except for horse no. 4 (described below). Diminished eating tended to coincide with fever, and appetence coincided with a return to normal temperature.

**Table 2:** Outcome for horses experimentally infected with *Venezuelan equine encephalitis virus* strains

Horse no.	Gender	Virus strain	Maximum temperature (°C)	Maximum viremia level^a^	Clinical outcome	Histopathologic findings
1	Male	OAX131	40.3	2.8	No disease	Perivascular cuffing, minimal encephalitis
2	Male	OAX131	40.2	1.4	No disease	Perivascular cuffing, minimal encephalitis
3	Male	OAX131	39.1	1.4	No disease	Perivascular cuffing, minimal encephalitis
4	Male	CPA201	40.8	2.4	Fatal encephalitis	Perivascular cuffing, lymphocytic meningitis, focal encephalitis
5	Male	CPA201	38.4	1.4	No disease	Normal
6	Female	CPA201	37.7	<0.6	No disease	Normal
7	Male	OAX142	38.2	<0.6	No disease	Normal
8	Female	OAX142	38.9	<0.6	No disease	Normal
9	Male	I-290–93	38.6	<0.6	No disease	Normal
10	Male	I-290–93	39.8	<0.6	No disease	Normal

^a^Titers expressed as log_10_ suckling mouse intracerebral lethal dose_50_/mL serum.

**Figure 2:**
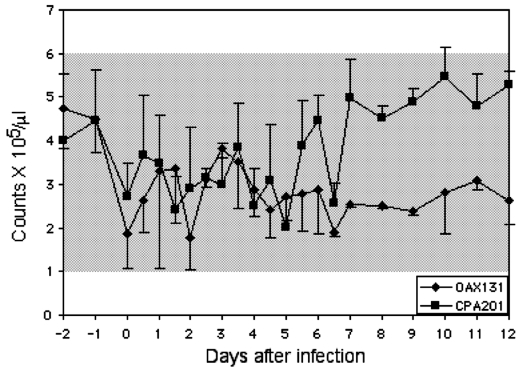
Mean platelet counts in horses infected with virus strains CPA201 and OAX131. Bars indicate standard deviations; shaded box indicates approximate normal values.

**Figure 3:**
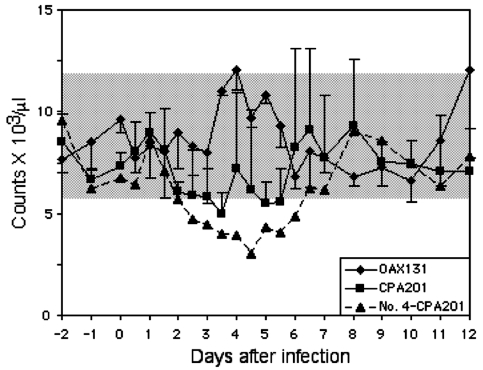
Mean leukocyte counts in horses infected with virus strains CPA201 and OAX131. Bars indicate standard deviations. Data for horse no. 4, in whom severe neurologic disease developed after infection with strain CPA201, are shown individually. Bars indicate standard deviations; shaded box indicates approximate normal values.

Platelet counts increased slightly after day 5 in horses infected with strain CPA201 (Figure 2). A modest leukopenia occurred from day 2 to day 6 in two of three animals infected with strain CPA201. Horse no. 4, infected with strain CPA201, developed severe neurologic disease and showed the greatest reduction in leukocyte counts (Figure 3). A slight leukocytosis was seen in animals infected with OAX131 from day 3 to day 5. Otherwise, most animals showed little evidence of changes in leukocyte counts. Hematocrit values did not drop (data not shown) as in most previous studies of equines infected with epizootic VEEV (*6*,*9*). Strains I-290–93 and OAX142 produced no apparent reduction in platelet counts (data not shown).

Horse no. 4 was the only animal that exhibited clinical signs of encephalitis. On day 6 postinoculation, this male appeared weak, and on day 8 it exhibited nervousness, anorexia, pendular head movements, marked incoordination of the extremities, penile relaxation, teeth grinding, muscular tremors, restlessness, dyspnea, head shaking, excessive sweating, movement of the ears in all directions, circular walking, and blindness. On day 9 this animal was more tranquil, with less sweating. It assumed an abnormal posture with its head resting on the wall, and blindness was evident because the horse began walking into various objects. On day 10, horse no. 4 showed incoordination, nervous ticks of the head, and complete blindness. By day 11 a weight loss was evident; on day 12 the horse was prostrate and was therefore killed.

### Viremia Levels

**Figure 4:**
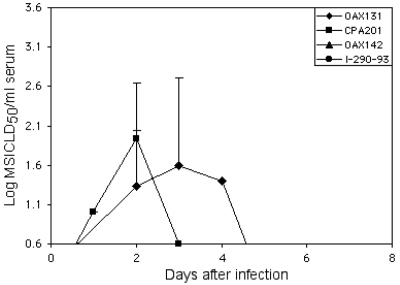
Mean viremia titers in horses infected with four different Mexican strains of subtype IE *Venezuelan equine encephalitis virus*. A log_10_ titer of 0.6 suckling mouse intracerebral lethal dose_50_ represents the maximum sensitivity of the assay. Bars indicate standard deviations.

Of 10 horses infected with VEEV strains from the two Mexican epizootics, none showed detectable viremia levels when serum specimens were assayed for plaques on Vero cells. When inoculation of newborn mice with serial dilutions was used, two of three horses infected with strain CPA201 and three horses infected with strain OAX131 demonstrated viremia with low titers on days 1–3 and 2–4, respectively (Figure 4). The other two VEEV strains (I-290–93 and OAX142) did not produce detectable viremia levels.

### Virus Isolation and Viral RNA Detection

**Figure 5:**
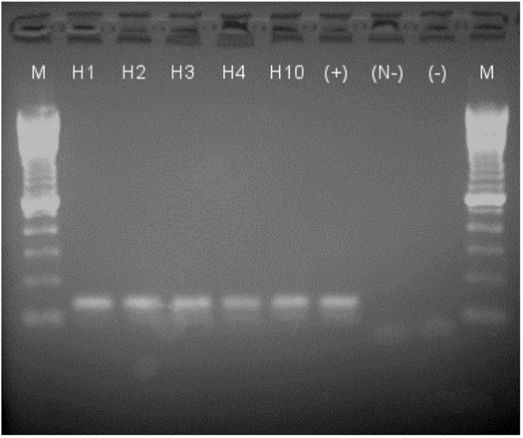
Results on 1.5% agarose gel of reverse tranferase–polymerase chain reaction (RT-PCR) products stained with ethidium bromide and imaged under UV light. M: 100-bp marker; H1–4, H10: horses 1–4 and 10; (+): positive control viral RNA, 1.8 x 10^2^ PFU amplified; (N-): negative control for nested PCR (-); negative control from single round RT-PCR. All horse samples and the positive control show a band at the expected size (134 bp), and negative controls show only the primers (below 100 bp).

Virus could not be isolated from brain tissues of any of the horses by inoculation of either Vero cells or baby mice. This was not unexpected because the animals were killed 12–15 days after infection, when the virus was presumably cleared by the immune response (neutralizing antibodies detected in all animals). Of the brain samples from horses 1–4 and 10 which were available for viral RNA detection by RT-PCR, all were positive (Figure 5). Sequences of the PCR amplicons were all identical to the parent strains, except for horse no. 4 infected with strain CPA201, which had a thymine to cytosine synonymous transition at nucleotide position 8562. Strain I-290–93 used to infect horse 10 had not been sequenced previously, but the sequence of the amplicon derived from horse 10 was identical to that of strain OAX131.

### Necropsy and Histopathologic Findings

**Figure 6:**
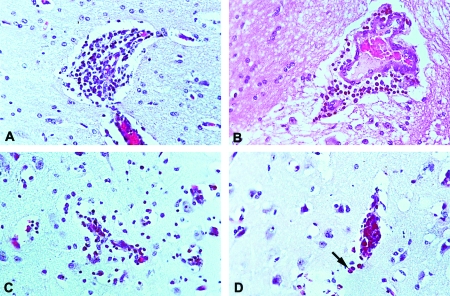
Representative histopathologic changes of brains of infected equines. A. Cerebrum, horse no. 3 (strain OAX131), showing perivascular cuff of lymphocytes (original magnification x200, hematoxylin and eosin [H&E] stain). B. Cerebrum, horse no. 4 (strain CPA201), showing perivascular cuff of lymphocytes (original magnification x200, H&E stain). C. Cerebral gray matter, horse no. 2 (strain OAX131), showing infiltration of mononuclear cells into brain parenchyma (original magnification x200, H&E stain), D. cerebral gray matter, horse no. 2, showing numerous eosinophils within perivascular infiltrate (arrow) (original magnification x200, H&E stain).

Gross pathologic lesions attributable to VEEV infection were not observed upon necropsy in any of the horses. Sections of brain from all 10 horses were examined. The fatally infected horse, no. 4, showed multifocal perivascular cuffing, lymphocytic meningitis, and focal encephalitis, characterized by focal neuronal necrosis and neuronophagia with associated microglial nodules (Figure 6B). Lesions were seen in gray and white matter of the cerebrum and cerebellum, but were not present in available sections of hippocampus or spinal cord. Samples of skeletal muscle and kidney from horse no. 10 showed no pathologic changes. Lymphoid tissue was not examined for any of the animals. All three horses infected with isolate OAX131 also showed evidence of meningitis with perivascular cuffing, and minimal encephalitis (Figure 6A). Animal no. 2 displayed more intense lesions, with numerous admixed eosinophils within leptomeningeal and perivascular infiltrates (Figures 6C and 6D).

## Discussion

### Equine Amplification and VEE Emergence

Previously described experimental infections of equines with epizootic subtype IAB and IC VEEV generally resulted in high rates of overt disease with high-titered viremia levels, often reaching 10^5-8^ suckling mouse intracerebral lethal dose 50% units (SMICLD_50_/mL serum) (*4*,*6*,*7*,*9*,*25*–*28*). This high-titered viremia, combined with the attractiveness of equines to many potential mosquito vectors and poor anti-mosquito defensive behavior, results in their high efficiency as amplification hosts for epizootic virus transmission. Equine case-fatality rates in these previous studies were generally approximately 50%, similar to those measured during natural epizootics. In contrast, enzootic VEEV belonging to subtypes ID (*6*,*9*), IE (*8*,*9*), II (Everglades virus) (*25*), III (Mucambo virus), and IV (Pixuna virus) (*29*) generally produces little or no clinical illness and viremia less than 10^5^ SMICLD_50_/mL serum after experimental equine infection. These enzootic viruses, including a subtype IE virus strain isolated near Veracruz near the Gulf Coast of Mexico, also have not generally been associated with equine disease in nature (*3*,*8*).

During 1969–1971, a VEE epizootic involving a subtype IAB strain spread northward from Guatemala along the Pacific Coast of Chiapas and Oaxaca States and then crossed into Veracruz State on the Gulf Coast before moving north to Texas (*5*). The reasons why the 1993 and 1996 Mexican outbreaks did not spread northward along the same route are unknown, but our studies suggest that the recent Mexican outbreaks were fundamentally different than those caused by subtype IAB and IC VEEV. The maximum viremia levels we measured in experimentally infected horses were approximately 1,000-fold lower than titers measured in previous studies using subtype IAB and IC VEEV strains from more extensive outbreaks (*4*,*6*,*7*,*9*,*25*–*28*,*30*). The titers we measured with the Mexican equine strains were also much lower than the infection thresholds for mosquitoes, as predicted by most experimental infections with VEEV (*31*–*34*). This finding indicates that equines probably did not serve as amplification hosts to support epizootic transmission cycles in Mexico during 1993 and 1996. This lack of equine amplification, which is believed to be a critical factor in VEE spread, probably limited the duration and scope of the 1993 and 1996 Mexican epizootics. The low-titered viremia level, even in the horse in whom fatal encephalitis developed, also indicates that the Mexican subtype IE epizootic viruses may be more neurotropic than other VEEV strains that apparently reach the central nervous system only after exceeding higher viremia titer thresholds (*4*,*6*,*9*).

Other possible explanations for sudden appearance of equine disease during the 1993 and 1996 outbreaks include increased transmission to horses by efficient amplification in other vertebrate hosts, or enhanced transmission by adaptation of local VEEV strains to equiphilic mosquito vectors. Although robust vector surveillance was not conducted during the 1993 and 1996 Mexican epizootics, rainfall patterns and anecdotal reports do not indicate unusually large mosquito populations. Vector incrimination and susceptibility testing are needed to assess the hypothesis that the Mexican epizootic strains are more infectious for mosquitoes and more readily transmitted than putative enzootic IE progenitors identified in phylogenetic studies (*15*,*16*).

### Equine Virulence of the Mexican VEEV Strains

In some respects, our results agreed with those of previous experimental studies of VEEV in equines. Infection with the Mexican VEEV strains resulted in mild leukopenia as has been reported (*9*). However, the mild hematologic abnormalities were more like those caused by enzootic VEEV strains than epizootic, subtype IAB and IC variants (*9*). In contrast to many VEE outbreaks attributed to subtype IAB and IC strains (*3*), the four Mexican epizootic virus strains we tested in horses produced an overall mortality rate of 10% but a case-fatality rate of 100%. These results also contrast with the reported case-fatality rates during the 1993 and 1996 Mexican outbreaks of 50% and 37%, respectively, and with the 1993 attack rate of 30% (*14*). The case-fatality rate differences probably reflect sampling error because our experimental infections produced disease in only one horse. Possible explanations for the discrepancy in apparent:inapparent ratios in our study versus data collected during the outbreaks include an underreporting of true inapparent infections during the 1993 epizootic, or phenotypic differences between the virus populations we inoculated into horses versus those circulating naturally during the epizootics. The latter possibility is discussed below.

Detection of VEEV RNA in the brain of five horses killed 12–14 days after infection, despite the absence of detectable infectious virus, was somewhat surprising. This result indicates that VEEV replicated in the brain of all horses, consistent with some pathologic lesions in the asymptomatic animals infected with strain OAX131 (Table 1). Persistence of viral RNA in the brain after the disappearance of infectious virus has also been reported for other alphaviruses (*35*).

Previous sequencing, rodent infection, and in vitro studies with these Mexican VEEV strains suggested that mixed populations of viruses may circulate in nature, represented by differences in amino acid composition and charge on the surface of the E2 envelope spike glycoprotein (*16*). Genomic sequencing studies of the four Mexican VEEV strains we studied revealed one amino acid difference: strain OAX142 has a Glu residue at position 117 of the E2 envelope glycoprotein, whereas the other three strains have Lys (*16*). The possibility that the Lys residue does not represent the wild-type sequence was considered previously because alphaviruses including VEEV are known to accumulate artifactual, positive charge amino acid changes in the E2 protein as the result of selection for binding to heparan sulfate on the surface of cells in culture (*36*). Because these changes reduce the virulence of alphaviruses in laboratory rodents, they can place into question the wild-type phenotype of VEEV strains.

However, our results further support the previous conclusion that the E2-117 Lys residue was present in natural VEEV isolates during the Mexican outbreaks as the consensus amino acid, but possibly in a mixed quasispecies population (*16*). The only strain that produced neurologic disease in our study was CPA201, which has a consensus Lys at E2-117. Strain OAX142, which has Glu-117, produced no detectable disease, viremia, or hematologic alterations. These results are not consistent with the hypothesis that the Lys at E2-117 resulted from heparan sulfate adaptation during RK cell passage, which would be expected to result in artificial attenuation (*36*). Because the Lys-117 mutation produces the small plaque phenotype characteristic of most epizootic, equine-virulent VEEV strains, it remains a potential virulence determinant worthy of further characterization by using cDNA clones and reverse genetics.
